# Methyl 4′-benzyl-2,2′-dimethyl-1,3-dioxo-2,3-dihydro-1*H*,4′*H*-spiro­[iso­quinoline-4,5′-oxazole]-4′-carboxyl­ate

**DOI:** 10.1107/S160053681101899X

**Published:** 2011-05-25

**Authors:** Hoong-Kun Fun, Ching Kheng Quah, Chengmei Huang, Haitao Yu

**Affiliations:** a X-ray Crystallography Unit, School of Physics, Universiti Sains Malaysia, 11800 USM, Penang, Malaysia; b School of Chemistry and Chemical Engineering, Nanjing University, Nanjing 210093, People’s Republic of China

## Abstract

In the isoquinoline ring system of the title mol­ecule, C_22_H_20_N_2_O_5_, the *N*-heterocyclic ring is in a half-boat conformation. The least-squares plane of the dioxa-2-aza­spiro ring [maximum deviation = 0.076 (1) Å] and forms a dihedral angle of 14.54 (4)° with the phenyl ring. In the crystal, mol­ecules are linked *via* inter­molecular C—H⋯O hydrogen bonds into layers parallel to (100).

## Related literature

For general background to and the potential biological activity of the title compound, see: Du *et al.* (2008 ▶); Chen *et al.* (2006 ▶); Mitchell *et al.* (1995 ▶, 2000 ▶); Galliford & Scheidt (2007 ▶); Badillo *et al.* (2010 ▶); Wang *et al.* (2010 ▶); Nair *et al.* (2002 ▶); Huang *et al.* (2011 ▶). For the stability of the temperature controller used in the data collection, see: Cosier & Glazer (1986 ▶). For standard bond-length data, see: Allen *et al.* (1987 ▶). For ring conformations, see: Cremer & Pople (1975 ▶). For related structures, see: Fun *et al.* (2011*a* ▶,*b* ▶,*c* ▶,*d* ▶).
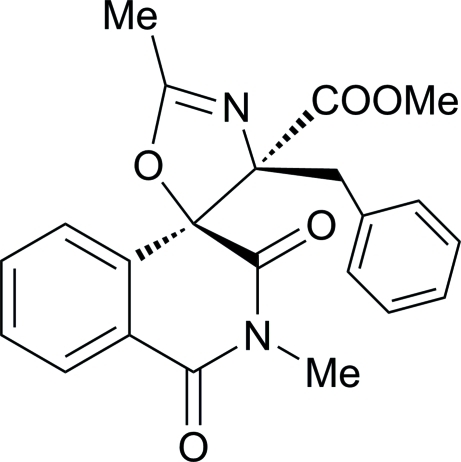


## Experimental

### 

#### Crystal data

C_22_H_20_N_2_O_5_
*M* *_r_* = 392.40
Triclinic, 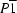

*a* = 8.6834 (7) Å
*b* = 11.1683 (9) Å
*c* = 11.3085 (9) Å
α = 100.638 (2)°
β = 106.347 (2)°
γ = 109.383 (2)°
*V* = 944.72 (13) Å^3^
*Z* = 2
Mo *K*α radiation
μ = 0.10 mm^−1^
*T* = 100 K
0.57 × 0.32 × 0.24 mm

#### Data collection

Bruker SMART APEXII DUO CCD area-detector diffractometer
Absorption correction: multi-scan (*SADABS*; Bruker, 2009 ▶) *T*_min_ = 0.937, *T*_max_ = 0.977
32434 measured reflections
8179 independent reflections
7354 reflections with *I* > 2σ(*I*)
*R*_int_ = 0.021

#### Refinement

*R*[*F* ^2^ > 2σ(*F* ^2^)] = 0.037
*wR*(*F* ^2^) = 0.109
*S* = 1.04
8179 reflections
265 parameters
H-atom parameters constrained
Δρ_max_ = 0.52 e Å^−3^
Δρ_min_ = −0.28 e Å^−3^

### 

Data collection: *APEX2* (Bruker, 2009 ▶); cell refinement: *SAINT* (Bruker, 2009 ▶); data reduction: *SAINT*; program(s) used to solve structure: *SHELXTL* (Sheldrick, 2008 ▶); program(s) used to refine structure: *SHELXTL*; molecular graphics: *SHELXTL*; software used to prepare material for publication: *SHELXTL* and *PLATON* (Spek, 2009 ▶).

## Supplementary Material

Crystal structure: contains datablocks global, I. DOI: 10.1107/S160053681101899X/rz2598sup1.cif

Structure factors: contains datablocks I. DOI: 10.1107/S160053681101899X/rz2598Isup2.hkl

Supplementary material file. DOI: 10.1107/S160053681101899X/rz2598Isup3.cml

Additional supplementary materials:  crystallographic information; 3D view; checkCIF report

## Figures and Tables

**Table 1:** Hydrogen-bond geometry (Å, °)

*D*—H⋯*A*	*D*—H	H⋯*A*	*D*⋯*A*	*D*—H⋯*A*
C15—H15*A*⋯O5^i^	0.93	2.50	3.4311 (11)	179
C19—H19*B*⋯O5^ii^	0.96	2.43	3.2594 (11)	145
C22—H22*A*⋯O3^iii^	0.96	2.53	3.2479 (8)	132

Symmetry codes: (i) 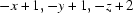; (ii) 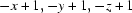; (iii) 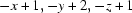.

**Fig. 1.**
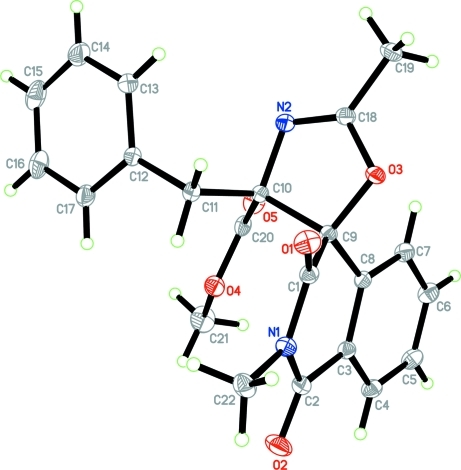
The molecular structure of the title compound showing 50% probability displacement ellipsoids for non-H atoms.

**Fig. 2.**
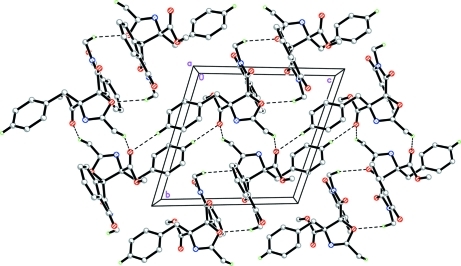
The crystal structure of the title compound, viewed along the a axis. H atoms not involved in hydrogen bonds (dashed lines) have been omitted for clarity.

## supplementary crystallographic information

### Comment

Isoquinoline-1,3,4-trione derivatives were reported to be a kind of small molecular inhibitor against caspase-3 which can promote apoptosis of the cells (Du *et al.*, 2008; Chen *et al.*, 2006). They can also attenuate apoptosis of neuronal cells induced by β-amyloid and have been reported to be redox mediators of photosystems I (Mitchell *et al.*, 2000; 1995). Spirocyclic oxindoles have emerged as attractive synthetic targets because of their prevalence in numerous natural products and their important biological activity (Galliford & Scheidt, 2007). Among them, the synthesis of spirooxindole oxazoles is of greatest interest (Badillo *et al.*, 2011; Wang *et al.*; 2010; Nair *et al.*, 2002). As a kind of analog of spiroindole oxazolines, spiroisoquinolineoxazolines have rarely been researched. Since a lot of bioactive natural products contain isoquinoline or oxazole rings, it is necessary to develop a methodology to construct such moieties. The title compound, which was derived from isoquinoline-1,3,4-trione and oxazoles (Huang *et al.*, 2011), may has a potential use in biochemical and pharmaceutical fields. Due to the importance of the isoquinoline-1,3,4-trione derivatives, we report in this paper the crystal structure of the title compound.

In the title racemic compound, Fig. 1, the isoquinoline ring system (N1/C1-C9) is not completely planar, the *N*-heterocyclic ring (N1/C1-C3/C8/C9) being distorted towards a half-boat conformation with atom C9 deviating by 0.216 (1) Å from the mean plane through the remaining atoms, puckering parameters (Cremer & Pople, 1975) Q = 0.3259 (7) Å, Θ =  112.68 (12)° and φ = 284.58 (13)°. The dioxa-2-azaspiro ring (N2/O3/C9/C10/C18) [maximum deviation of 0.076 (1) Å for atom C9] is inclined at a dihedral angle of  14.54 (4)° with the phenyl ring (C12-C17). Bond lengths (Allen *et al.*, 1987) and angles are within normal ranges and comparable to those found in related structures (Fun *et al.*, 2011*a*,*b*,*c*, *d*).

In the crystal structure (Fig. 2), molecules are linked *via* intermolecular C15–H15A···O5, C19–H19B···O5 and C22–H22A···O3 hydrogen bonds (Table 1) into two-dimensional layers parallel to (100).

### Experimental

The title compound was the main product from the acid-catalyzed transformation of the photocycloadduct of isoquinoline-1,3,4-trione and 4-benzyl-5-methoxy-2-methyloxazole. The compound was purified by flash column chromatography with ethyl acetate/petroleum ether (1:4 *v*/*v*) as eluents. X-ray quality crystals of the title compound were obtained from slow evaporation of an acetone and petroleum ether solution (1:5 *v*/*v*). M.p. 451-453 K.

### Refinement

All H atoms were positioned geometrically and refined using a riding model with C–H = 0.93 - 0.97 Å and *U*_iso_(H) = 1.2 or 1.5 *U*_eq_(C). A rotating-group model was applied for the methyl groups. The highest residual electron density peak and the deepest hole are located at 0.62 and 0.59 Å from C16, respectively.

### Crystal data

**Table:**

C_22_H_20_N_2_O_5_	*Z* = 2
*M**_r_* = 392.40	*F*(000) = 412
Triclinic, *P*1	*D*_x_ = 1.379 Mg m^−^^3^
Hall symbol: -P 1	Mo *K*α radiation, λ = 0.71073 Å
*a* = 8.6834 (7) Å	Cell parameters from 9462 reflections
*b* = 11.1683 (9) Å	θ = 2.4–37.5°
*c* = 11.3085 (9) Å	µ = 0.10 mm^−^^1^
α = 100.638 (2)°	*T* = 100 K
β = 106.347 (2)°	Block, colourless
γ = 109.383 (2)°	0.57 × 0.32 × 0.24 mm
*V* = 944.72 (13) Å^3^	

### Data collection

**Table:**

Bruker SMART APEXII DUO CCD area-detector diffractometer	8179 independent reflections
Radiation source: fine-focus sealed tube	7354 reflections with *I* > 2σ(*I*)
graphite	*R*_int_ = 0.021
φ and ω scans	θ_max_ = 35.0°, θ_min_ = 2.4°
Absorption correction: multi-scan (*SADABS*; Bruker, 2009)	*h* = −14→14
*T*_min_ = 0.937, *T*_max_ = 0.977	*k* = −15→17
32434 measured reflections	*l* = −18→18

### Refinement

**Table:**

Refinement on *F*^2^	Primary atom site location: structure-invariant direct methods
Least-squares matrix: full	Secondary atom site location: difference Fourier map
*R*[*F*^2^ > 2σ(*F*^2^)] = 0.037	Hydrogen site location: inferred from neighbouring sites
*wR*(*F*^2^) = 0.109	H-atom parameters constrained
*S* = 1.04	*w* = 1/[σ^2^(*F*_o_^2^) + (0.0618*P*)^2^ + 0.1885*P*] where *P* = (*F*_o_^2^ + 2*F*_c_^2^)/3
8179 reflections	(Δ/σ)_max_ = 0.001
265 parameters	Δρ_max_ = 0.52 e Å^−^^3^
0 restraints	Δρ_min_ = −0.28 e Å^−^^3^

### Special details

**Table:**

Experimental. The crystal was placed in the cold stream of an Oxford Cryosystems Cobra open-flow nitrogen cryostat (Cosier & Glazer, 1986) operating at 100.0 (1) K.
Geometry. All esds (except the esd in the dihedral angle between two l.s. planes) are estimated using the full covariance matrix. The cell esds are taken into account individually in the estimation of esds in distances, angles and torsion angles; correlations between esds in cell parameters are only used when they are defined by crystal symmetry. An approximate (isotropic) treatment of cell esds is used for estimating esds involving l.s. planes.
Refinement. Refinement of F^2^ against ALL reflections. The weighted R-factor wR and goodness of fit S are based on F^2^, conventional R-factors R are based on F, with F set to zero for negative F^2^. The threshold expression of F^2^ > 2sigma(F^2^) is used only for calculating R-factors(gt) etc. and is not relevant to the choice of reflections for refinement. R-factors based on F^2^ are statistically about twice as large as those based on F, and R- factors based on ALL data will be even larger.

### Fractional atomic coordinates and isotropic or equivalent isotropic displacement parameters (Å^2^)

**Table:**

	*x*	*y*	*z*	*U*_iso_*/*U*_eq_	
O1	0.42744 (6)	0.99506 (5)	0.62495 (5)	0.01754 (9)	
O2	1.02131 (7)	1.14156 (6)	0.79359 (6)	0.02414 (11)	
O3	0.38562 (6)	0.75687 (5)	0.48448 (4)	0.01382 (8)	
O4	0.79722 (6)	0.82611 (5)	0.84267 (5)	0.01722 (9)	
O5	0.66059 (8)	0.60673 (6)	0.73207 (5)	0.02126 (10)	
N1	0.72403 (7)	1.06714 (5)	0.71929 (5)	0.01359 (9)	
N2	0.34962 (7)	0.62897 (5)	0.61741 (5)	0.01486 (9)	
C1	0.55724 (8)	0.97088 (6)	0.64348 (6)	0.01269 (10)	
C2	0.87944 (8)	1.05358 (6)	0.72190 (6)	0.01481 (10)	
C3	0.86167 (8)	0.93183 (6)	0.63044 (6)	0.01287 (10)	
C4	1.01135 (8)	0.92673 (7)	0.61052 (6)	0.01605 (11)	
H4A	1.1205	0.9972	0.6568	0.019*	
C5	0.99607 (9)	0.81563 (7)	0.52111 (7)	0.01776 (11)	
H5A	1.0950	0.8117	0.5070	0.021*	
C6	0.83222 (9)	0.71001 (7)	0.45264 (7)	0.01777 (11)	
H6A	0.8221	0.6365	0.3918	0.021*	
C7	0.68345 (8)	0.71352 (6)	0.47458 (6)	0.01523 (10)	
H7A	0.5750	0.6419	0.4299	0.018*	
C8	0.69821 (7)	0.82499 (6)	0.56378 (5)	0.01185 (9)	
C9	0.54340 (7)	0.82960 (6)	0.59671 (5)	0.01139 (9)	
C10	0.50493 (7)	0.75045 (6)	0.69900 (5)	0.01179 (9)	
C11	0.46381 (8)	0.82409 (6)	0.80912 (6)	0.01338 (10)	
H11A	0.5604	0.9106	0.8561	0.016*	
H11B	0.3589	0.8385	0.7715	0.016*	
C12	0.43613 (8)	0.74497 (6)	0.90251 (6)	0.01339 (10)	
C13	0.27481 (9)	0.64000 (7)	0.87237 (7)	0.02070 (13)	
H13A	0.1812	0.6222	0.7971	0.025*	
C14	0.25276 (12)	0.56155 (8)	0.95414 (8)	0.02710 (16)	
H14A	0.1448	0.4916	0.9328	0.033*	
C15	0.39115 (13)	0.58721 (8)	1.06747 (8)	0.02569 (15)	
H15A	0.3768	0.5337	1.1210	0.031*	
C16	0.55074 (11)	0.69343 (9)	1.09983 (7)	0.02314 (14)	
H16A	0.6432	0.7124	1.1762	0.028*	
C17	0.57309 (9)	0.77188 (7)	1.01826 (6)	0.01805 (11)	
H17A	0.6805	0.8431	1.0411	0.022*	
C18	0.29331 (8)	0.64261 (6)	0.50621 (6)	0.01369 (10)	
C19	0.13538 (8)	0.54771 (7)	0.39220 (6)	0.01767 (11)	
H19A	0.0703	0.4749	0.4165	0.027*	
H19B	0.1709	0.5142	0.3247	0.027*	
H19C	0.0627	0.5927	0.3616	0.027*	
C20	0.65977 (8)	0.71589 (6)	0.75831 (6)	0.01428 (10)	
C21	0.95690 (10)	0.80855 (10)	0.89813 (8)	0.02757 (16)	
H21A	1.0476	0.8923	0.9566	0.041*	
H21B	0.9932	0.7771	0.8302	0.041*	
H21C	0.9360	0.7446	0.9443	0.041*	
C22	0.73534 (9)	1.19379 (7)	0.79492 (6)	0.01796 (11)	
H22A	0.6594	1.2240	0.7407	0.027*	
H22B	0.8542	1.2593	0.8272	0.027*	
H22C	0.6998	1.1809	0.8663	0.027*	

### Atomic displacement parameters (Å^2^)

**Table:**

	*U*^11^	*U*^22^	*U*^33^	*U*^12^	*U*^13^	*U*^23^
O1	0.01526 (19)	0.0181 (2)	0.0226 (2)	0.00961 (17)	0.00716 (17)	0.00806 (17)
O2	0.0138 (2)	0.0238 (3)	0.0220 (2)	0.00140 (18)	0.00273 (17)	−0.00360 (19)
O3	0.01078 (17)	0.01457 (19)	0.01226 (17)	0.00306 (14)	0.00146 (14)	0.00387 (14)
O4	0.01345 (19)	0.0230 (2)	0.01511 (19)	0.00824 (17)	0.00345 (15)	0.00683 (17)
O5	0.0283 (3)	0.0210 (2)	0.0243 (2)	0.0163 (2)	0.0132 (2)	0.01167 (19)
N1	0.0134 (2)	0.0122 (2)	0.0139 (2)	0.00491 (16)	0.00435 (16)	0.00305 (16)
N2	0.0148 (2)	0.0125 (2)	0.0140 (2)	0.00247 (17)	0.00536 (17)	0.00256 (16)
C1	0.0130 (2)	0.0130 (2)	0.0130 (2)	0.00544 (18)	0.00502 (18)	0.00536 (18)
C2	0.0130 (2)	0.0161 (2)	0.0131 (2)	0.00428 (19)	0.00430 (18)	0.00351 (19)
C3	0.0115 (2)	0.0142 (2)	0.0129 (2)	0.00490 (18)	0.00465 (17)	0.00471 (18)
C4	0.0124 (2)	0.0181 (3)	0.0190 (3)	0.0061 (2)	0.00679 (19)	0.0073 (2)
C5	0.0166 (2)	0.0195 (3)	0.0229 (3)	0.0097 (2)	0.0110 (2)	0.0091 (2)
C6	0.0194 (3)	0.0168 (3)	0.0215 (3)	0.0092 (2)	0.0113 (2)	0.0063 (2)
C7	0.0154 (2)	0.0143 (2)	0.0167 (2)	0.00607 (19)	0.00724 (19)	0.00404 (19)
C8	0.0115 (2)	0.0131 (2)	0.0121 (2)	0.00527 (18)	0.00493 (17)	0.00502 (17)
C9	0.0099 (2)	0.0121 (2)	0.0110 (2)	0.00388 (17)	0.00275 (16)	0.00363 (17)
C10	0.0120 (2)	0.0119 (2)	0.0116 (2)	0.00449 (17)	0.00453 (17)	0.00413 (17)
C11	0.0149 (2)	0.0137 (2)	0.0129 (2)	0.00626 (19)	0.00623 (18)	0.00452 (18)
C12	0.0143 (2)	0.0141 (2)	0.0123 (2)	0.00542 (19)	0.00596 (18)	0.00399 (18)
C13	0.0189 (3)	0.0198 (3)	0.0159 (3)	0.0000 (2)	0.0070 (2)	0.0030 (2)
C14	0.0346 (4)	0.0170 (3)	0.0238 (3)	0.0000 (3)	0.0162 (3)	0.0046 (2)
C15	0.0435 (4)	0.0207 (3)	0.0256 (3)	0.0168 (3)	0.0223 (3)	0.0135 (3)
C16	0.0285 (3)	0.0333 (4)	0.0191 (3)	0.0195 (3)	0.0126 (3)	0.0145 (3)
C17	0.0164 (2)	0.0244 (3)	0.0145 (2)	0.0084 (2)	0.0062 (2)	0.0075 (2)
C18	0.0115 (2)	0.0130 (2)	0.0147 (2)	0.00366 (18)	0.00504 (18)	0.00219 (18)
C19	0.0126 (2)	0.0172 (3)	0.0165 (2)	0.0031 (2)	0.00291 (19)	−0.0004 (2)
C20	0.0162 (2)	0.0179 (3)	0.0131 (2)	0.0089 (2)	0.00716 (19)	0.00792 (19)
C21	0.0180 (3)	0.0424 (5)	0.0250 (3)	0.0168 (3)	0.0040 (2)	0.0140 (3)
C22	0.0221 (3)	0.0137 (2)	0.0161 (2)	0.0076 (2)	0.0056 (2)	0.0022 (2)

### Geometric parameters (Å, °)

**Table:**

O1—C1	1.2153 (7)	C10—C20	1.5294 (8)
O2—C2	1.2168 (8)	C10—C11	1.5562 (8)
O3—C18	1.3707 (8)	C11—C12	1.5167 (9)
O3—C9	1.4316 (7)	C11—H11A	0.9700
O4—C20	1.3417 (8)	C11—H11B	0.9700
O4—C21	1.4459 (9)	C12—C13	1.3952 (9)
O5—C20	1.2033 (8)	C12—C17	1.3998 (9)
N1—C1	1.3841 (8)	C13—C14	1.3954 (11)
N1—C2	1.3999 (8)	C13—H13A	0.9300
N1—C22	1.4682 (8)	C14—C15	1.3920 (13)
N2—C18	1.2719 (8)	C14—H14A	0.9300
N2—C10	1.4607 (8)	C15—C16	1.3873 (12)
C1—C9	1.5212 (8)	C15—H15A	0.9300
C2—C3	1.4813 (9)	C16—C17	1.3937 (10)
C3—C8	1.3958 (8)	C16—H16A	0.9300
C3—C4	1.3972 (9)	C17—H17A	0.9300
C4—C5	1.3899 (10)	C18—C19	1.4839 (9)
C4—H4A	0.9300	C19—H19A	0.9600
C5—C6	1.3943 (10)	C19—H19B	0.9600
C5—H5A	0.9300	C19—H19C	0.9600
C6—C7	1.3936 (9)	C21—H21A	0.9600
C6—H6A	0.9300	C21—H21B	0.9600
C7—C8	1.3934 (9)	C21—H21C	0.9600
C7—H7A	0.9300	C22—H22A	0.9600
C8—C9	1.5064 (8)	C22—H22B	0.9600
C9—C10	1.6217 (8)	C22—H22C	0.9600
			
C18—O3—C9	106.97 (5)	C12—C11—H11B	109.3
C20—O4—C21	115.40 (6)	C10—C11—H11B	109.3
C1—N1—C2	124.19 (5)	H11A—C11—H11B	108.0
C1—N1—C22	116.77 (5)	C13—C12—C17	118.43 (6)
C2—N1—C22	118.93 (5)	C13—C12—C11	120.52 (6)
C18—N2—C10	107.79 (5)	C17—C12—C11	121.01 (6)
O1—C1—N1	122.07 (6)	C12—C13—C14	120.55 (7)
O1—C1—C9	121.60 (5)	C12—C13—H13A	119.7
N1—C1—C9	116.00 (5)	C14—C13—H13A	119.7
O2—C2—N1	120.27 (6)	C15—C14—C13	120.51 (7)
O2—C2—C3	122.69 (6)	C15—C14—H14A	119.7
N1—C2—C3	116.99 (5)	C13—C14—H14A	119.7
C8—C3—C4	120.54 (6)	C16—C15—C14	119.32 (7)
C8—C3—C2	120.73 (5)	C16—C15—H15A	120.3
C4—C3—C2	118.72 (5)	C14—C15—H15A	120.3
C5—C4—C3	119.56 (6)	C15—C16—C17	120.25 (7)
C5—C4—H4A	120.2	C15—C16—H16A	119.9
C3—C4—H4A	120.2	C17—C16—H16A	119.9
C4—C5—C6	119.90 (6)	C16—C17—C12	120.90 (7)
C4—C5—H5A	120.0	C16—C17—H17A	119.6
C6—C5—H5A	120.0	C12—C17—H17A	119.6
C7—C6—C5	120.63 (6)	N2—C18—O3	118.47 (5)
C7—C6—H6A	119.7	N2—C18—C19	127.74 (6)
C5—C6—H6A	119.7	O3—C18—C19	113.79 (5)
C8—C7—C6	119.60 (6)	C18—C19—H19A	109.5
C8—C7—H7A	120.2	C18—C19—H19B	109.5
C6—C7—H7A	120.2	H19A—C19—H19B	109.5
C7—C8—C3	119.75 (5)	C18—C19—H19C	109.5
C7—C8—C9	121.41 (5)	H19A—C19—H19C	109.5
C3—C8—C9	118.72 (5)	H19B—C19—H19C	109.5
O3—C9—C8	109.49 (5)	O5—C20—O4	124.54 (6)
O3—C9—C1	107.97 (5)	O5—C20—C10	125.43 (6)
C8—C9—C1	112.93 (5)	O4—C20—C10	110.02 (5)
O3—C9—C10	102.22 (4)	O4—C21—H21A	109.5
C8—C9—C10	113.35 (5)	O4—C21—H21B	109.5
C1—C9—C10	110.23 (4)	H21A—C21—H21B	109.5
N2—C10—C20	110.16 (5)	O4—C21—H21C	109.5
N2—C10—C11	109.10 (5)	H21A—C21—H21C	109.5
C20—C10—C11	109.43 (5)	H21B—C21—H21C	109.5
N2—C10—C9	102.88 (4)	N1—C22—H22A	109.5
C20—C10—C9	109.71 (4)	N1—C22—H22B	109.5
C11—C10—C9	115.34 (5)	H22A—C22—H22B	109.5
C12—C11—C10	111.59 (5)	N1—C22—H22C	109.5
C12—C11—H11A	109.3	H22A—C22—H22C	109.5
C10—C11—H11A	109.3	H22B—C22—H22C	109.5
			
C2—N1—C1—O1	−165.85 (6)	C18—N2—C10—C20	−126.15 (5)
C22—N1—C1—O1	10.31 (9)	C18—N2—C10—C11	113.70 (6)
C2—N1—C1—C9	20.58 (8)	C18—N2—C10—C9	−9.25 (6)
C22—N1—C1—C9	−163.26 (5)	O3—C9—C10—N2	12.51 (5)
C1—N1—C2—O2	−178.23 (6)	C8—C9—C10—N2	−105.22 (5)
C22—N1—C2—O2	5.68 (9)	C1—C9—C10—N2	127.10 (5)
C1—N1—C2—C3	4.38 (9)	O3—C9—C10—C20	129.73 (5)
C22—N1—C2—C3	−171.70 (5)	C8—C9—C10—C20	12.00 (6)
O2—C2—C3—C8	170.61 (6)	C1—C9—C10—C20	−115.68 (5)
N1—C2—C3—C8	−12.07 (9)	O3—C9—C10—C11	−106.16 (5)
O2—C2—C3—C4	−10.41 (10)	C8—C9—C10—C11	136.11 (5)
N1—C2—C3—C4	166.91 (6)	C1—C9—C10—C11	8.43 (7)
C8—C3—C4—C5	1.63 (9)	N2—C10—C11—C12	66.89 (6)
C2—C3—C4—C5	−177.35 (6)	C20—C10—C11—C12	−53.70 (6)
C3—C4—C5—C6	−0.36 (10)	C9—C10—C11—C12	−177.96 (5)
C4—C5—C6—C7	−1.16 (10)	C10—C11—C12—C13	−80.98 (7)
C5—C6—C7—C8	1.40 (10)	C10—C11—C12—C17	96.54 (7)
C6—C7—C8—C3	−0.13 (9)	C17—C12—C13—C14	−1.85 (10)
C6—C7—C8—C9	−176.13 (6)	C11—C12—C13—C14	175.73 (6)
C4—C3—C8—C7	−1.38 (9)	C12—C13—C14—C15	0.33 (12)
C2—C3—C8—C7	177.58 (6)	C13—C14—C15—C16	1.25 (12)
C4—C3—C8—C9	174.72 (5)	C14—C15—C16—C17	−1.28 (11)
C2—C3—C8—C9	−6.31 (8)	C15—C16—C17—C12	−0.26 (11)
C18—O3—C9—C8	109.09 (5)	C13—C12—C17—C16	1.82 (10)
C18—O3—C9—C1	−127.60 (5)	C11—C12—C17—C16	−175.74 (6)
C18—O3—C9—C10	−11.36 (5)	C10—N2—C18—O3	2.44 (7)
C7—C8—C9—O3	−33.67 (7)	C10—N2—C18—C19	−177.79 (6)
C3—C8—C9—O3	150.29 (5)	C9—O3—C18—N2	6.70 (7)
C7—C8—C9—C1	−154.01 (6)	C9—O3—C18—C19	−173.11 (5)
C3—C8—C9—C1	29.96 (7)	C21—O4—C20—O5	3.34 (9)
C7—C8—C9—C10	79.73 (7)	C21—O4—C20—C10	−175.41 (5)
C3—C8—C9—C10	−96.30 (6)	N2—C10—C20—O5	7.11 (8)
O1—C1—C9—O3	28.45 (8)	C11—C10—C20—O5	127.05 (6)
N1—C1—C9—O3	−157.94 (5)	C9—C10—C20—O5	−105.46 (7)
O1—C1—C9—C8	149.65 (6)	N2—C10—C20—O4	−174.16 (5)
N1—C1—C9—C8	−36.74 (7)	C11—C10—C20—O4	−54.21 (6)
O1—C1—C9—C10	−82.44 (7)	C9—C10—C20—O4	73.27 (6)
N1—C1—C9—C10	91.17 (6)		

### Hydrogen-bond geometry (Å, °)

**Table:**

*D*—H···*A*	*D*—H	H···*A*	*D*···*A*	*D*—H···*A*
C15—H15A···O5^i^	0.93	2.50	3.4311 (11)	179
C19—H19B···O5^ii^	0.96	2.43	3.2594 (11)	145
C22—H22A···O3^iii^	0.96	2.53	3.2479 (8)	132

Symmetry codes: (i) −*x*+1, −*y*+1, −*z*+2; (ii) −*x*+1, −*y*+1, −*z*+1; (iii) −*x*+1, −*y*+2, −*z*+1.
